# A Case Series on Minimally Invasive Upper Arm Contouring: Efficacy of Combined Renuvion Helium Plasma and Icoone Multi‐Micro Alveolar Stimulation for Fat Reduction

**DOI:** 10.1002/ccr3.72243

**Published:** 2026-03-16

**Authors:** Mohammad Ali Nilforoushzadeh, Alireza Jafarzadeh, Tannaz Fakhim, Masoumeh Mohamadi, Fereshteh Salarvand, Farnaz Parvaresh, Niloufar Najar Nobari

**Affiliations:** ^1^ Skin and Stem Cell Research Center Tehran University of Medical Sciences Tehran Iran; ^2^ Skin Repair Research Center, Jordan Dermatology and Hair Transplantation Center Tehran Iran

**Keywords:** fat reduction, helium plasma, Icoone, minimally invasive treatments, multi micro alveolar stimulation, Renuvion, upper arm contouring

## Abstract

The combination of Renuvion helium plasma and Icoone Multi Micro Alveolar Stimulation is an effective, minimally invasive approach for reducing upper arm fat and improving skin texture. This synergistic treatment provides aesthetic improvements with minimal risk and recovery time.

## 1 Introduction

Upper arm fat reduction and body contouring are highly sought‐after aesthetic goals, with patients increasingly opting for minimally invasive treatments to improve their body image. The upper arm, in particular, is a commonly targeted area for fat reduction due to its tendency to retain fat even with regular physical activity and healthy lifestyle choices. Traditional fat reduction techniques, such as liposuction, often carry risks and extended recovery times, prompting a surge in demand for safer, non‐surgical alternatives [1, 2].

Minimally invasive body contouring technologies have gained significant attention in recent years. Among these, Renuvion helium plasma and Icoone Multi Micro Alveolar Stimulation (MMAS) have shown promise in treating stubborn fat and improving skin tone and texture [3, 4]. Renuvion employs a unique helium plasma‐based system to deliver controlled energy beneath the skin, targeting subdermal fat while simultaneously promoting collagen production and skin tightening [3]. Icoone, on the other hand, uses mechanical massage and suction to activate connective tissue, enhancing lymphatic drainage, improving circulation, and reducing localized fat [4].

Although both treatments have demonstrated effectiveness individually [4, 5, 6], there is limited research on their combined use in upper arm contouring. This case series aims to evaluate the efficacy and safety of a combined protocol using Renuvion and Icoone for reducing upper arm fat, improving skin appearance, and providing enhanced aesthetic results. The combination of these two minimally invasive technologies could provide a synergistic effect, potentially offering a novel solution for patients seeking non‐surgical alternatives to traditional fat‐reduction methods.

The present study, involving three adult female patients, seeks to address this gap in the literature by assessing the clinical outcomes of this combined treatment. The findings from this case series have the potential to contribute significantly to the growing body of knowledge on minimally invasive body contouring and could help guide future research in this area.

## 2 Case History/Examination

Three adult female patients, aged 52, 45, and 47, were selected for a minimally invasive arm contouring treatment. None of the patients had a history of significant medical conditions or previous treatments aimed at reducing arm fat. All participants had no prior cosmetic interventions or medical treatments such as liposuction. Each patient sought improvement in the appearance of their upper arms and expressed a desire for a minimally invasive procedure to reduce excess arm fat.

Assessment of arm composition was carried out using the InBody analyzer, which allowed for precise measurements of fat mass before and after the treatment protocol. A detailed physical examination confirmed the presence of redundant skin and localized fat in the upper arm region.

## 3 Methods

The study included adult female patients aged between 45 and 52 years, with localized upper arm fat and no significant underlying medical conditions or previous interventions for fat reduction. All participants were selected based on their desire for a minimally invasive procedure to improve the appearance of their upper arms. Patients with contraindications for Minimally Invasive procedures or those with a history of significant medical issues such as cardiovascular disease or diabetes were excluded from the study.

A standardized minimally invasive treatment protocol was employed, which included Renuvion and Icoone therapies. The treatment parameters for Renuvion were set as follows: Power: 80, helium flow rate: 1.5 L/min, total energy delivered: 18 kJ, and suction pressure: 150 mmHg. Renuvion was applied to both arms using subdermal techniques.

Circumference measurements were taken at standardized anatomical landmarks using a non‐stretch tape with the arm relaxed by the patient's side. To ensure reproducibility and accuracy, triplicate measurements were taken by a single trained operator, and the mean value was used for analysis.

Following the Renuvion treatment, Icoone was applied to all patients. The Icoone system utilizes Multi Micro Alveolar Stimulation (MMAS), a technique involving mechanical massage and suction to activate connective tissue at a microscopic level. The protocol involved two phases. The first phase, known as the base treatment, lasted 30 min and aimed at promoting general lymphatic drainage and improving circulation. Using Robotwins handpieces, the treatment was applied to the entire body or target areas, reducing edema and promoting detoxification. The second phase, the focus treatment, lasted between 25 and 35 min per session, concentrating on the upper arm region. The power level was set at 8, with rhythmic wave frequencies at 13 Hz and 10 Ip.

This phase helped reduce localized fat and further enhanced the results of the Renuvion treatment. Six Icoone sessions were performed, one each week following the Renuvion procedure.

The Renuvion system uses helium plasma to deliver controlled energy beneath the skin, which heats the subdermal fat tissue, promoting fat reduction while stimulating collagen production. This process leads to skin tightening and improves overall skin texture. The Icoone system uses mechanical massage combined with suction to activate the connective tissue at a microscopic level. This promotes lymphatic drainage, improves circulation, and facilitates fat reduction by stimulating the body's natural processes.

Each patient was assessed at baseline before the treatment and 1 week after the final session of Icoone treatment. Follow‐up evaluations included the measurement of arm fat mass using the InBody analyzer, and both Physician's Global Assessment (PGA) and Patient's Global Assessment (PGA) were recorded at the start and end of the treatment. This systematic follow‐up ensured that the changes observed in fat mass and aesthetic appearance were tracked over time and allowed for accurate assessment of the treatment's effectiveness.

1. Physician's Global Assessment (PGA):

The PGA was used by the treating physician to assess the overall outcome of the treatment. Based on visual inspection and clinical judgment, the physician assigned a score reflecting the improvement in upper arm fat mass and aesthetic appearance. The scoring scale was as follows:

0: No improvement.

1: Poor.

2: Fair.

3: Good.

4: ExcellentThe PGA was performed at the beginning and end of the treatment period to monitor changes in the patient's condition.

2 Patient's Global Assessment (PGA):

The Patient's Global Assessment was completed by the patients to evaluate their satisfaction with the results of the treatment. This assessment focused on the patient's perception of aesthetic changes and overall improvement in upper arm appearance. The scale used was:

0: Very dissatisfied.

1: Dissatisfied.

2: Neutral.

3: Satisfied.

4: Very satisfiedPatients provided their evaluations both before and after the treatment to capture any changes in their satisfaction levels.

Additionally, arm fat mass was measured objectively using the InBody analyzer both before the treatment and 1 week after the final Icoone session. This provided a quantitative measure of fat reduction in conjunction with the subjective evaluations from both the physician and the patient.

To ensure the reliability and accuracy of the measurements, all circumference measurements of the upper arm were taken using a non‐stretch tape at standardized anatomical landmarks. Three measurements were taken by a trained operator, and the mean value was used for the analysis. The Physician's Global Assessment (PGA) was performed by the treating physician, while the Patient's Global Assessment (PGA) was provided by the patients to assess their satisfaction with the treatment outcomes. This dual assessment method, along with the objective measurements from the InBody analyzer, ensured comprehensive and standardized evaluation of the treatment's impact.

## 4 Conclusion and Results

**FIGURE 1:**
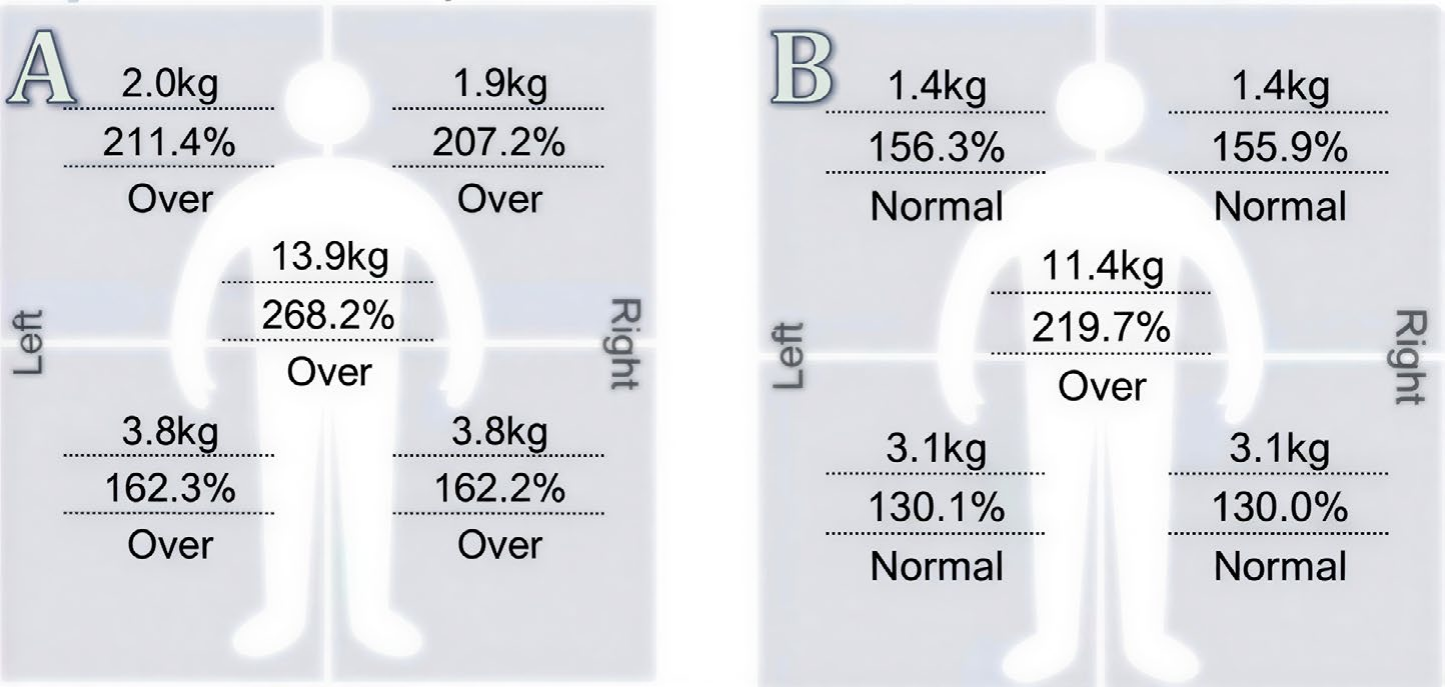
Segmental Fat Analysis showing the fat distribution before (A) and after (B) treatment for Patient 1. Analysis (A) reveals higher fat mass across the left arm before treatment, while analysis (B) shows improvements in fat distribution and overall body composition in the left arm after treatment.

**FIGURE 2:**
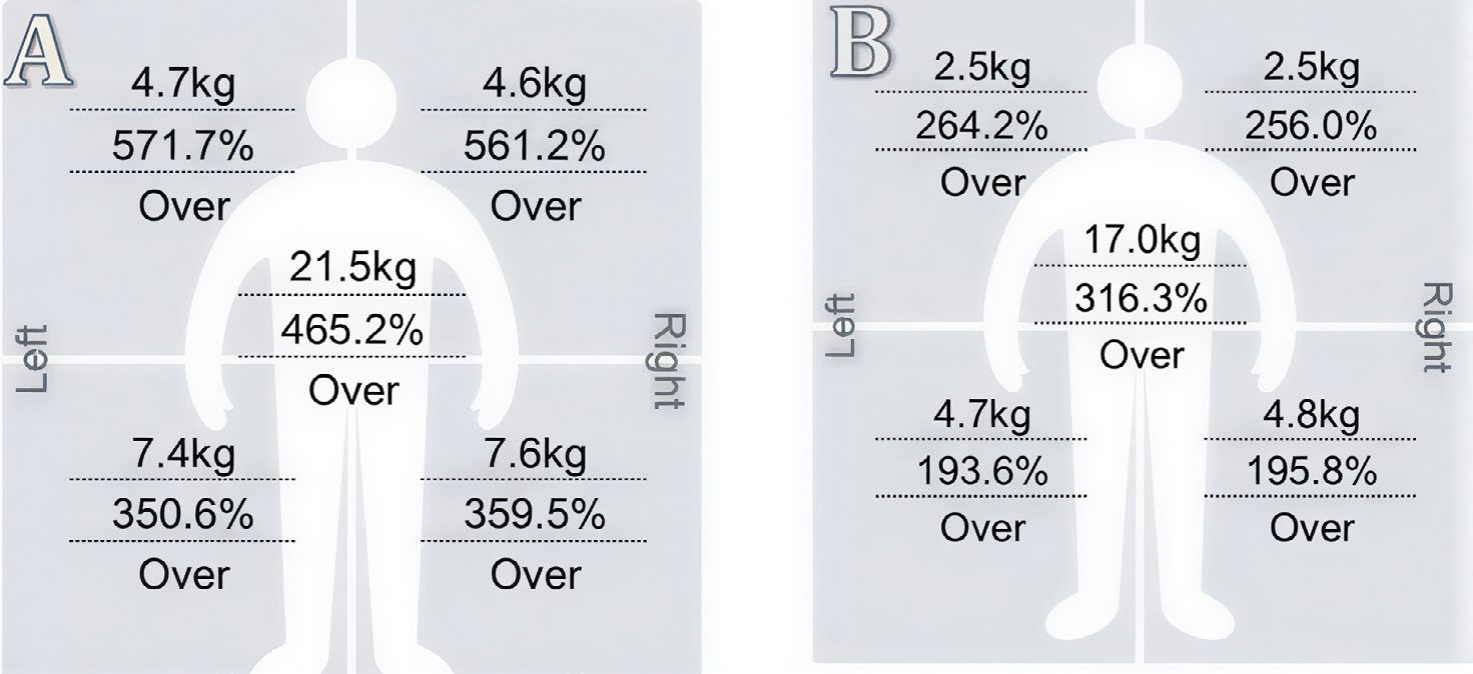
Segmental Fat Analysis showing the fat distribution before (A) and after (B) treatment for Patient 2. In analysis (A), the left arm exhibits a higher fat mass, whereas in analysis (B), improvements in fat reduction and overall body contouring are visible in the left arm post‐treatment.

**FIGURE 3:**
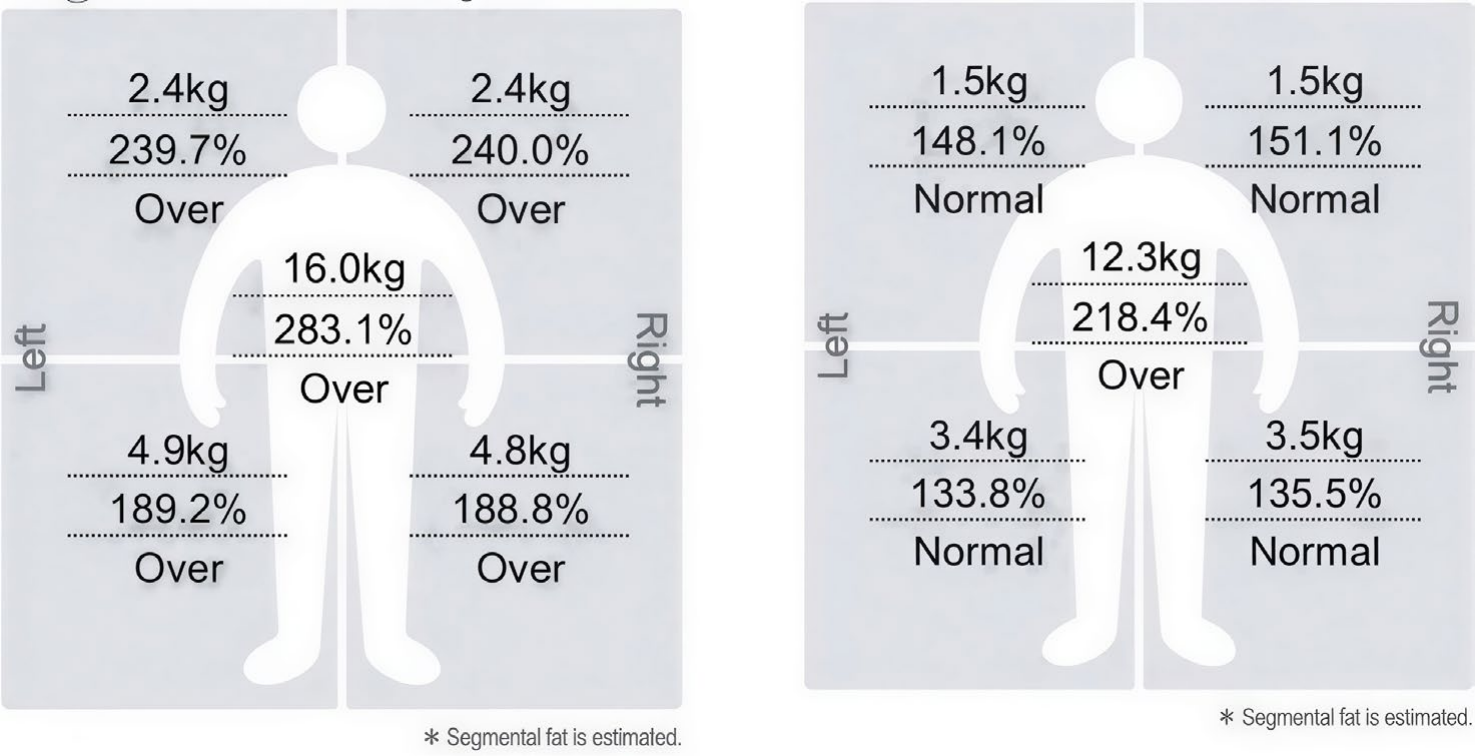
Segmental Fat Analysis showing the fat distribution before (A) and after (B) treatment for Patient 3. In analysis (A), the right arm exhibits a higher fat mass, while in analysis (B), there is a reduction in fat and improvement in the overall contour of the right arm post‐treatment.

**FIGURE 4:**
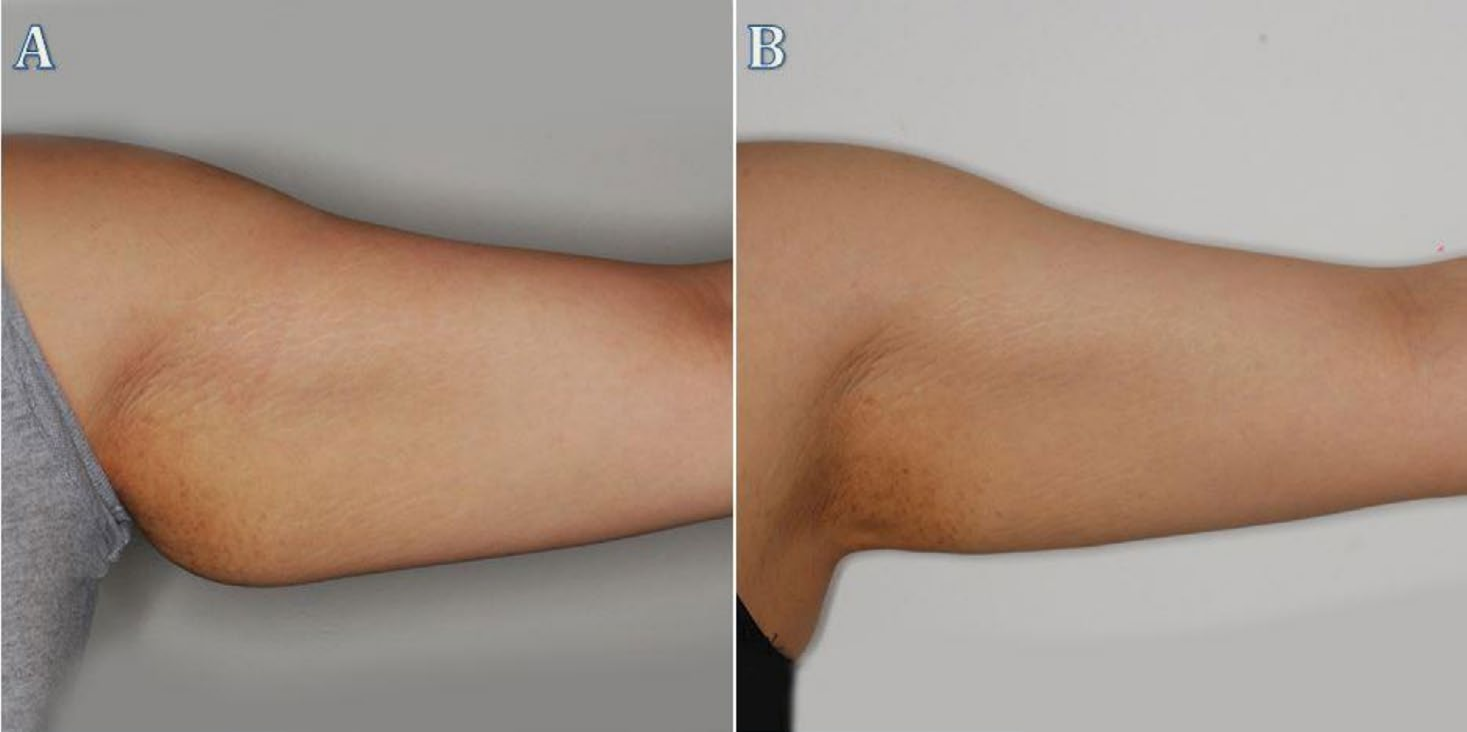
Clinical image showing the left arm of Patient 1 before (A) and after (B) treatment. In image (A), excess fat and skin laxity are evident, while in image (B), fat reduction and improvement in the arm contour are visible post‐treatment.

**FIGURE 5:**
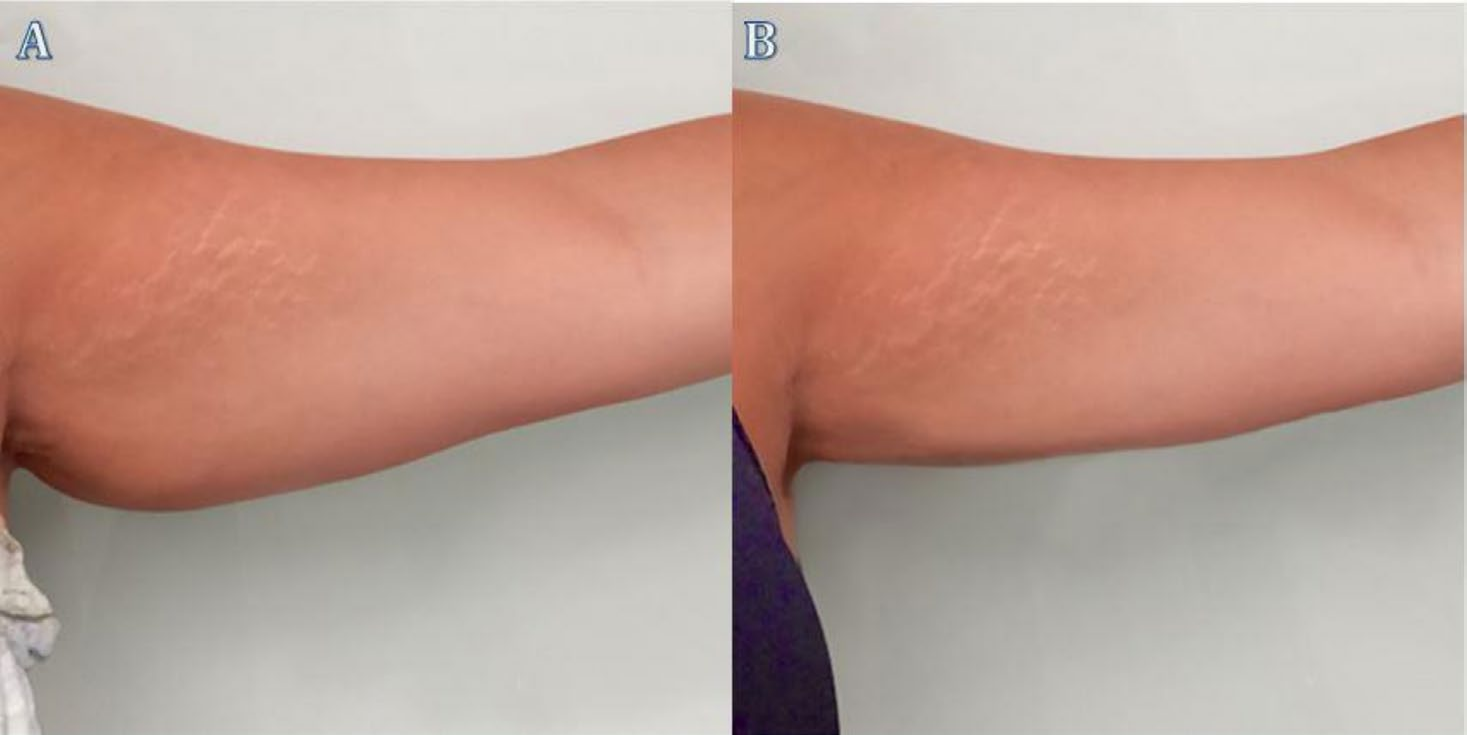
Clinical image showing the left arm of Patient 2 before (A) and after (B) treatment. In image (A), the left arm displays noticeable fat and skin laxity, along with visible stretch marks. In image (B), there is a visible reduction in fat and improvement in skin tone and texture post‐treatment.

**FIGURE 6:**
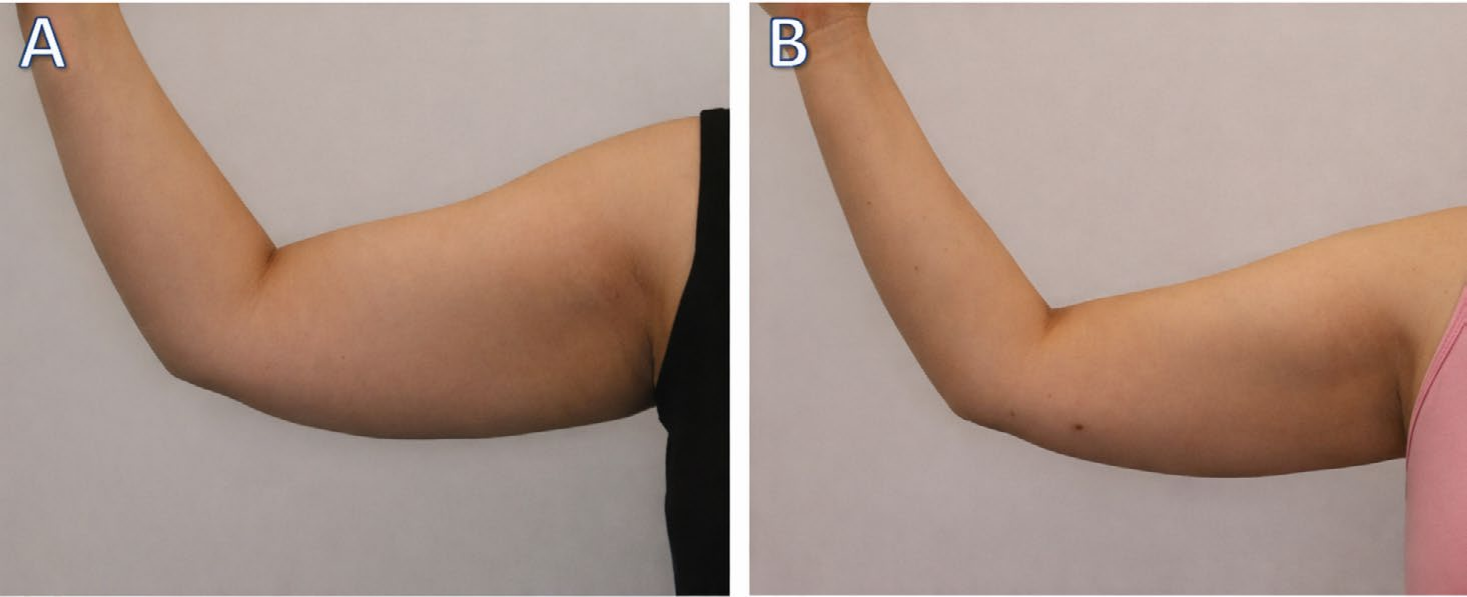
Clinical image of the right arm before (A) and after (B) treatment, showing a noticeable reduction in fat and improvement in arm contour. In image (A), the arm shows visible fat accumulation, while in image (B), the arm appears more toned with reduced fat mass and improved skin appearance post‐treatment.

All three patients showed a reduction in upper arm fat mass following the treatment protocol (Figures 1, 2, 3, 4, 5, 6), with the results as follows:

- Patient 1 (52 years old): Reduction from 2 to 1.4 kg (0.6 kg decrease).
- Patient 2 (45 years old): Reduction from 4.7 to 2.5 kg (2.2 kg decrease).
- Patient 3 (47 years old): Reduction from 2.4 to 1.5 kg (0.9 kg decrease).

The average reduction in arm fat mass was 1.5 kg across all patients. No adverse events were reported, and the patients expressed high satisfaction with the aesthetic improvements.

Additionally, changes in the Physician's Global Assessment (PGA) and Patient's Global Assessment (PGA) scores were observed in all three patients (Table 1):

- Patient 1 showed improvement in both assessments. The Physician's PGA score increased from 1 (poor) to 3 (good), while the Patient's PGA score improved from 0 (very dissatisfied) to 3 (satisfied).
- Patient 2 demonstrated an increase in the Physician's PGA from 0 (no improvement) to 4 (excellent), while the Patient's PGA also rose from 1 (dissatisfied) to 3 (satisfied), indicating a positive response to the treatment.
- Patient 3 experienced improvements in both evaluations, with the Physician's PGA rising from 1 (poor) to 3 (good) and the Patient's PGA improving from 1 (dissatisfied) to 4 (very satisfied).

**TABLE 1:** Physician's and Patient's Global Assessment (PGA) scores for three patients.

Patient	Physician's PGA (start)	Physician's PGA (end)	Patient's PGA (start)	Patient's PGA (end)
Patient 1	1	3	0	3
Patient 2	0	4	1	3
Patient 3	1	3	1	4

*Note:* This table illustrates the changes in both Physician's PGA and Patient's PGA scores from the start to the end of the treatment for three patients, reflecting improvements in the treatment outcomes.

These findings suggest that the combination of Renuvion and Icoone is an effective and safe minimally invasive protocol for reducing upper arm fat mass, with favorable outcomes reflected in both physician and patient assessments.

## 5 Discussion

The combined application of Renuvion helium plasma and Icoone Multi Micro Alveolar Stimulation (MMAS) demonstrated improvements in upper arm contouring in this case series. All three patients experienced reductions in localized fat and improvements in skin texture and tone. These results are in line with previous studies that have highlighted the efficacy of Renuvion for minimally invasive body contouring [7, 8]. Renuvion utilizes a unique combination of helium plasma and radiofrequency energy to target subdermal fat and promote skin tightening, thereby improving the overall aesthetic appearance [2, 3]. Furthermore, Icoone employs a mechanical massage technique combined with suction, which enhances lymphatic drainage, improves circulation, and facilitates lipolysis, contributing to fat reduction and skin rejuvenation [9].

In addition to its ability to reduce fat, Renuvion stimulates collagen production, which leads to improved skin elasticity and tightening [10]. This has been well‐documented in the literature, with several studies reporting enhanced skin quality following Renuvion treatment, particularly in post‐surgical patients [5, 6, 10]. The synergistic effect of combining Renuvion and Icoone may offer a more comprehensive solution by addressing both fat reduction and skin laxity, thus providing superior outcomes in terms of body contouring.

The combination of Renuvion and Icoone targets both fat reduction and skin tightening. The helium plasma from Renuvion heats the subdermal fat tissue, leading to lipolysis (breakdown of fat), while simultaneously stimulating collagen production, which enhances skin elasticity. This mechanism is supported by studies showing that plasma energy can effectively stimulate collagen synthesis. Similarly, Icoone activates the lymphatic system through mechanical massage, which improves circulation and facilitates fat reduction. These combined treatments target both fat cells and the skin's underlying connective tissue, leading to improved body contouring and skin rejuvenation [9, 10].

When compared to other minimally invasive body contouring technologies such as cryolipolysis, laser lipolysis, and radiofrequency (RF) treatments, the combination of Renuvion and Icoone offers several advantages.

Cryolipolysis is a minimally invasive technique that uses controlled cold temperatures to induce apoptosis in fat cells, leading to a reduction in fat volume. While it is effective for localized fat reduction, it does not directly address issues such as skin laxity or tone, which may limit its overall aesthetic benefits. The treatment typically requires multiple sessions to achieve noticeable results, and side effects like temporary numbness, tingling, or delayed fat loss may occur. These factors can influence patient satisfaction, as the desired results may take time to fully manifest [11, 12].

Laser lipolysis utilizes laser energy to liquefy fat cells, leading to fat reduction. While it can improve skin appearance through thermal tightening, it often carries a higher risk of adverse effects, such as burns or skin irritation, and typically requires longer recovery times compared to Renuvion. These factors may limit its appeal for patients seeking quicker recovery and minimal risk of complications [13].

Radiofrequency (RF) treatments use radiofrequency energy to heat the skin, stimulating collagen production and promoting skin tightening. While effective for improving skin tone and elasticity, RF treatments generally do not lead to significant fat reduction, making them less effective for contouring areas with stubborn fat. Additionally, RF devices often require multiple sessions to achieve optimal results, which can extend the treatment timeline for patients seeking faster, more noticeable outcomes [14].

In contrast, the combination of Renuvion and Icoone addresses both fat reduction and skin tightening in a single protocol, potentially offering better results with fewer treatment sessions and shorter recovery times. This dual approach may lead to superior patient satisfaction, as it targets both aesthetic concerns—excess fat and skin laxity—simultaneously.

The safety and tolerability of Renuvion and Icoone treatments are well‐established in the literature [2, 3, 4, 5]. In this study, all patients tolerated the treatments well, with no significant adverse events reported.

Patients also expressed high satisfaction with the results, noting improved skin texture, reduced fat mass, and enhanced overall appearance. This aligns with findings from studies on other minimally invasive treatments, such as laser lipolysis and cryolipolysis, which report moderate to high satisfaction levels [11, 12, 13]. However, the combination of Renuvion and Icoone offers the additional benefit of skin tightening, which is often not achieved with other fat‐reduction methods alone.

This study has several limitations that should be considered when interpreting the results. Firstly, the lack of a control group limits our ability to conclusively attribute the observed outcomes to the intervention alone, as there are no comparisons to rule out the effects of natural variation or other potential factors. Secondly, the absence of standardized concomitant interventions introduces variability in the treatment process, which may affect the consistency and generalizability of the findings. Lastly, the failure to account for potential confounders further limits the ability to draw definitive conclusions regarding the efficacy of the devices used. These factors should be addressed in future studies to enhance the scientific rigor of the findings.

## Author Contributions

**Mohammad Ali Nilforoushzadeh:** conceptualization, supervision, writing – review and editing. **Alireza Jafarzadeh:** conceptualization, supervision, visualization, writing – original draft, writing – review and editing. **Tannaz Fakhim:** visualization, writing – original draft. **Masoumeh Mohamadi:** investigation, writing – review and editing. **Fereshteh Salarvand:** investigation, methodology. **Farnaz Parvaresh:** investigation. **Niloufar Najar Nobari:** methodology, visualization.

## Funding

The authors have nothing to report.

## Disclosure

Transparency Declaration: The authors affirm that the manuscript is honest, accurate, and transparent. No important aspect of the study has been omitted.

## Consent

Written informed consent was obtained from all patients, and their privacy was maintained in accordance with ethical standards. All patient photos included in this manuscript have been anonymized to ensure patient confidentiality. Based on the guidelines of the local medical research ethics committee, registration of this study is not required.

## Conflicts of Interest

The authors declare no conflicts of interest.

## Data Availability

The data that support the findings of this study are available from the corresponding author upon reasonable request.
